# Prevalence of occupational injuries and knowledge of availability and utilization of post exposure prophylaxis among health care workers in Singida District Council, Singida Region, Tanzania

**DOI:** 10.1371/journal.pone.0201695

**Published:** 2018-10-25

**Authors:** Lucina Kimaro, Juma Adinan, Damian J. Damian, Bernard Njau

**Affiliations:** 1 Singida District Council, Singida, Tanzania; 2 Kilimanjaro Christian Medical University College, Moshi Tanzania; 3 Community Health Department, Kilimanjaro Christian Medical Centre, Moshi, Tanzania; 4 School of Assistant Medical Officer (AMO), KCMC. Moshi, Tanzania; Massachusetts General Hospital, UNITED STATES

## Abstract

**Background:**

Infection with Human Immunodeficiency Virus is a serious public health problem that threatens the lives of many people including health care workers. Health care workers are frequently exposed to occupational hazards throughout their careers. Health care workers are at risk of being infected by the virus when caring for patients in health care facilities. Utilization of HIV Post-exposure Prophylaxis (HIV PEP) is very vital once an individual is exposed.

**Aim:**

The aim of this study is to determine the prevalence of occupational exposure, knowledge of, availability and utilization of post exposure prophylaxis among health care workers in Singida District Council, Tanzania.

**Methods:**

A descriptive cross sectional study was conducted from April to May 2013. Health care workers actively treating patients were enrolled from 18 heath facilities in Singida District Council. Data were collected using a self-administered questionnaire, and analysed using Stata version 12.

**Results:**

Out of 239 participants, slightly more than half, 124 (52%) had inadequate overall knowledge of HIV PEP. Of the 239, 121(50.6%) participants experienced occupational exposure. Two leading types of exposure were blood splash 57(47.1%) and needle stick injuries 45 (37.2%),respectively. Among the 121 exposed participants, 83(68.6%) reported the exposure incident, 91(75.2%) had an HIV test, 32 (26.4%), started HIV PEP after testing, 28 (23.1%), completed HIV PEP, and 65 (53.7%) had a follow-up HIV test. About two thirds (159/239), of participants reported that HIV PEP services were available at the time the study was conducted, and 49 (20.5%), reported daily access to HIV PEP service.

**Conclusion:**

The prevalence of occupational exposure among health care workers is high with low utilization of HIV PEP. The majority of healthcare workers had inadequate knowledge of HIV PEP. The findings highlight the need to improve the level of knowledge of HIV PEP and utilization of PEP among this at-high-risk-group in Singida.

## Introduction

HIV/AIDS is a major public health problem globally costing the lives of many people including Health care workers (HCWs)[1]. Each day a large number of HCWs in the world suffer accidental exposures to blood-borne pathogens[1]. It is estimated that 3 million percutaneous exposures to blood or other body fluids occur in health care settings annually[2]. Ninety percent of occupational exposures around the world occur in developing countries. Occupational exposure may lead to infection by bloodborne pathogens, including HIV. The average risk of seroconversion to HIV after a single percutaneous exposure to HIV-infected blood is 0.1–0.3%. However, the risk of transmission through exposure to blood splash alone does not confer risk. Splash of blood or body fluids to a mucous membrane does have risk, but less than a needle stick[3][4][5][6].

HIV PEP is an emergency medical response given as soon as possible to reduce the risk of HIV infection after potential exposure. Despite the high prevalence of occupational exposure to HIV[3][7][4][8][5], the utilization of HIV PEP is low[3][4][5]. Thousands of HCWs worldwide, experience occupational exposure due to blood and other body fluids or tissue while performing their duties. HCWs become vulnerable as they deal with the patients whose sero status is either known or unknown. PEP utilization can reduce the risk of acquiring HIV at the work site, by 81% [3], if it is initiated as soon as possible and not later than 72 hours following exposure[6].

Tanzania is also facing challenges of HIV occupational exposure and the low utilization of HIV PEP among HCWs [9–11]. Prevalence of HIV in Tanzania is around 5.1% [12]. The risk of becoming infected on the job is a threat to everyone working in healthcare facilities.

Lack of knowledge among HCWs increases the risk of acquiring HIV infection at the workplace. Evidence suggests a lack of information about PEP in many healthcare settings. For example, a study in Ethiopia showed that 83.9% of HCWs had inadequate knowledge of PEP for HIV. Additionally, 81.6% of those exposed, never used PEP, and 33.8% did not use PEP because of the lack of information about PEP[13].

Low awareness of HIV PEP use and complicated procedures to obtain the HIV PEP are the main issues that contribute to low utilization of HIV PEP. Limited information on the level of awareness of HCWs concerning HIV PEP used at work sites and about transmission risk in resource-constrained settings poses the largest burden of HIV infection. Underreporting of exposure and other potential occupational exposures are widely reported[8].

Singida District Council reported three cases of HCWs with needle stick injuries at their workplaces in 2011. However, information regarding utilization of HIV PEP among the three reported cases was missing. This observed gap on proper handling of occupational exposures underlined the importance of this study to estimate the magnitude of the problem in this setting.

This study therefore was conducted to determine the prevalence of occupational exposure, availability and utilization of PEP for HIV among HCWs actively dealing with patients in Singida District Council.

## Methods

### Study design

This was a cross sectional study conducted from April to May 2013

### Study area

The study was conducted in Singida District Council, one of six districts in Singida Region. Singida District Council has a total population of 225,521 (male = 111,772 (49.6%); female = 113,749 (50.4%); average household size of 5.4; population growth rate of 2.3 based on the 2012 National Census.[14]. The study area has 12,164 square kilometres; the lowest wealth quintiles was 33.3 compared to the highest wealth quintiles of 6.4 (Gini coefficient = 47.7). Small scale business and farming are the main economic activities conducted by the Singida District population.

There are a total of 59 health facilities, out of which, three (5.1%) hospitals are owned by Faith Based Organisations (FBOs), five (8.5%) are government owned health centres, and 51(86.4%) government owned dispensaries. During the study period, out of 59 health facilities, 38 (64.4%) were providing HIV services and, 10/38 (26. 3%), were providing PEP services.

In 2012, the HIV prevalence in Singida Region was 3.3%; 4.5% for females, and 1.8% for males.

### Study population

We selected all cadres of HCWs actively dealing with patients from 18 public and private health facilities. HCWs attending patients either in wards or at the clinics or those who are handling, processing and analyzing specimens are referred to here as HCWs actively dealing with patients. HCWs involved in the study were: medical officers (MDs), nurses (i.e. registered nurses (RN)/enrolled nurses (ENs), assistant medical officers (AMOs), clinical officers (COs), clinical assistants (CAs), laboratory personnel, and medical attendants (MAs). Types of health facilities involved were; hospitals (n = 3; 16.6%), health centers (n = 5; 27.7%) and dispensaries (n = 10; 55.5%).

### Sampling technique

Hospitals and health centers were purposively selected and provided 133 (n = 44 per each hospital) and 75 (n = 15 per health centre) participants respectively.

Ten dispensaries were systematically selected from 30 dispensaries providing HIV services. A systematic sampling procedure to select the 10 dispensaries was as follows: 1) a list of all 30 dispensaries providing HIV services was used as a sampling frame, 2) A sampling interval was calculated using the following formula (n/N), whereby n = the number of the 10 dispensaries to be selected/ N = total number of all 30 dispensaries. A sampling interval of 1/3 was used to select the 10 dispensaries out of all 30 dispensaries. To select the first dispensary a lottery method was used. Three pieces of paper numbered 1 to 3 were put into a box and selected. The name of dispensary in the sampling frame corresponding to the number picked from the box was selected first to be included in the study. Consecutive dispensaries where selected using the sampling interval of three, until all 10 dispensaries where selected. Dispensaries provided 30 participants (n = 3 per dispensary).

### Data collection method and tools

Data were collected through a self-administered structured questionnaire administered by the first author [LK] and two trained research assistants. Questionnaires were adopted from two settings [15,16], and modified to address the objectives of this study. The questionnaire was available in both English and Swahili. English version of the S1 Questionnaire was used for data collection. The questionnaire was in paper-based format and participants were offered the questionnaires once they consented and were asked to return the filled questionnaire in a closed envelope to a specified office within 24 to 72 hours. Research Assistants collected the filled questionnaires daily during the study period.

The level of knowledge about HIV PEP was assessed using 10 questions. An example of a knowledge question was: “What are the regimens used for HIV PEP according to risk levels? Expected response was: 1 = low risk-dual therapy (two drugs); 4 = high risk-triple therapy (three drugs) Availability of PEP was assessed by using 4 questions. An example question was: “Is HIV PEP always available in this facility?”. Expected response was: 1 = Yes; 2 = No. Utilization of HIV PEP was assessed by using 5 questions. An example question was: “Did you start HIV PEP after HIV testing?”. Expected response was: 1 = Yes; 2 = No. Pretesting of the tool was done among ten HCWs from four health facilities, which were not included in the study. Any ambiguity and uncertainty of specific questions were modified before starting data collection.

### Scoring of knowledge and utilisation of PEP for HIV

Ten questions assessed the level of knowledge on HIV PEP. The expected maximum score was 10 points and minimum score was 0. The overall knowledge was summarized into 2 categories-adequate and inadequate. Participants who scored seven points and above were categorized having adequate knowledge and those who scored below seven points as having inadequate knowledge. For each individual question in the knowledge assessement the responses were categorized into knowledgeable and not knowledgeable if the participant provide correct and incorrect response respectively.

### Data analysis

Data was double-entered in Epi Data version 3.1 then transferred to Stata 12 SE for cleaning, processing and analysis. Continuous data were presented using measures of central tendencies with their respective measures of dispersion. Categorical variables were summarized into percentages. Results were summarized and presented into frequencies and tables respectively.

### Ethical approval

Ethical approval to conduct this study was obtained from Kilimanjaro Christian Medical University College Ethics committee. Permission to conduct this study in the health facilities was obtained from the Singida District Medical Officer and the Singida District Executive Director. The aim and objectives of the study were explained to the participants and written informed consent to participate in the study was obtained. The study involved adult healthcare providers. Participants’ records were made anonymous for confidentiality.

## Results

### Characteristics of respondents

A total of 239 HCWs participated in this study, a response rate of 100%. More than half of the participants were females 148 (61.9%). The mean age (±SD) of participants at enrolment was 39.1 (±10.7) years. More than half of the study participants were married 143 (59.8%); with certificate level of education 149 (62.3%); nurses 120 (50.2%); 55.6% working in FBO facilities, and 101(42.3%), had 15+ years of service. See Table 1.

**Table 1:** Background characteristics of the studyd participants (n = 239).

Characteristics	n (%)
***Age (years)*:**	
≤ 20	7 (2.9)
21–40	123 (51.5)
41–60	106 (44.4)
61 +	3 (1.3)
*Mean (±SD)*, *years*	*39*.*1±10*.*7*
***Sex*:**	
Male	91 (38.1)
Female	148 (61.9)
***Marital status*:**	
Married	143 (59.8)
Single	68 (28.5)
Others	28 (11.7)
***Educational level*:**	
Certificate	149(62.3)
Diploma/Advanced diploma	81 (33.9)
Degree	9 (3.8)
***Designation*:**	
Medical Officers	10 (4.2)
Assistant Medical Officers	13 (5.4)
Clinical Officers	33 (13.8)
Nurses(Registered/enrolled)	120 (50.2)
Laboratory personnel	17 (7.1)
Medical Attendants	46 (19.3)
***Years of experience*:**	
≤ 5	67 (28.0)
5–9	51 (21.3)
10–14	20 (8.4)
15 +	101 (42.3)
***Ownership of health facility*:**	
Government	106 (44.4)
Faith Based Organisation	133 (55.6)
***Department*:**	
Pediatrics	39 (16.3)
Medical	54 (22.6)
Surgical	21 (8.8)
Obstetrics & Gynecology	19 (7.9)
Reproductive and Child Health	55 (23.0)
Laboratory	26 (10.9)
Others	25 (10.5)

### Prevalence of occupational exposure among HCWs

More than half 121 (50.6%) of the participants reported to have ever had occupational exposures. Fifty seven (47.1%) reported exposure to blood splash and 45 (37.2%) had needle stick. Of 121, 61 (50.4%) reported exposure to have occurred in the past one year. Table 2 shows these findings.

**Table 2:** Prevalence of occupational exposure among study participants.

Variables	n (%)
*Ever had occupational exposures(n = 239)*	
Yes	121 (50.6)
No	118 (49.4)
*Type of exposures (n = 121)*:	
Blood splash	57 (47.1)
Needle stick injury	45 (37.2)
Mucous splash	14 (11.6)
Others	5 (4.1)
*Time frame of the occurrence (n = 121)*:	
Within 3 months	37 (30.6)
Within 6 months	23(19.0)
In the past one year	61 (50.4)

### Level of knowledge on HIV post exposure prophylaxis among HCWs

Slightly more than half, 124 (51.9%) of HCWs had inadequate overall knowledge of HIV PEP. Overall mean (**±**SD) score of the level of knowledge on HIVPEP was 4.4 (±1.4) points (ranging from 0 to 7 points). Most participants were knowledgeable of the drugs used for a high risk 176 (73.6%), definition of HIV PEP (69.5%), criteria for offering certain PEP regimen (68.2%), drugs used for a low risk (59.4%), and type of HIV PEP regimens according to the level of risk (55.2%). Most participants were not knowledgeable on the importance of using HIV PEP (84.1%), the duration of HIV PEP medication (71%), and the steps to be followed after having occupational exposure (62%) See Table 3.

**Table 3:** Level of knowledge of HIV post exposure prophylaxis among HCWs (n = 239).

Items	Level of Knowledge n (%)	
	Knowledgeable	Not knowledgeable
Knowledge of definition of HIV PEP	73 (30.5)	166 (69.5)
Knowledge of the source of occupational injury	141 (59.0)	98 (41.0)
Knowledge of the importance of using HIV PEP	201 (84.1)	38 (15.9)
Knowledge of the criteria for offering certain PEP regimen	76 (31.8)	163 (68.2)
Knowledge of the duration of HIV PEP medication	169 (70.7)	70 (29.3)
Knowledge of when to start HIV PEP medication	130 (54.4)	109 (45.6)
Knowledge of the type of HIV PEP regimens according to level of risk	107 (44.8)	132 (55.2)
Knowledge of the drugs used for a low risk	97 (40.6)	142 (59.4)
Knowledge of the drugs used for a high risk	63 (26.4)	176 (73.6)
Knowledge of the steps to be followed after having occupational exposure	148 (61.9)	91 (38.1)
Overall level of knowledge about HIV PEP	115 (48.1)	124 (51.9)

### Availability of HIV post exposure prophylaxis among HCWs

Among all the participants in this study, 179 (74.9%) reported that the HIV PEP was available at their work place. Out of 179 respondents, more than half 104 (58.1%) reported to have a person available to administer the HIV PEP, 24 hours a day. About two thirds (159) of participants reported that HIV PEP was always available at the health facility and only 49 (20.5%) reported daily accessibility to HIV PEP service as shown in Table 4.

**Table 4:** Availability of HIV post exposure prophylaxis among HCWs (n = 239).

Variables	n (%)
HIV PEP always available at the facility	159 (66.5)
HIV PEP availability at the respective department	179 (74.9)
HIV PEP accessible daily	49 (20.5)
There is a person to administer HIV PEP 24 hours a day (n = 179)	104 (58.1)

### Utilization rate of HIV post exposure prophylaxis among HCWs

**Fig 1:**
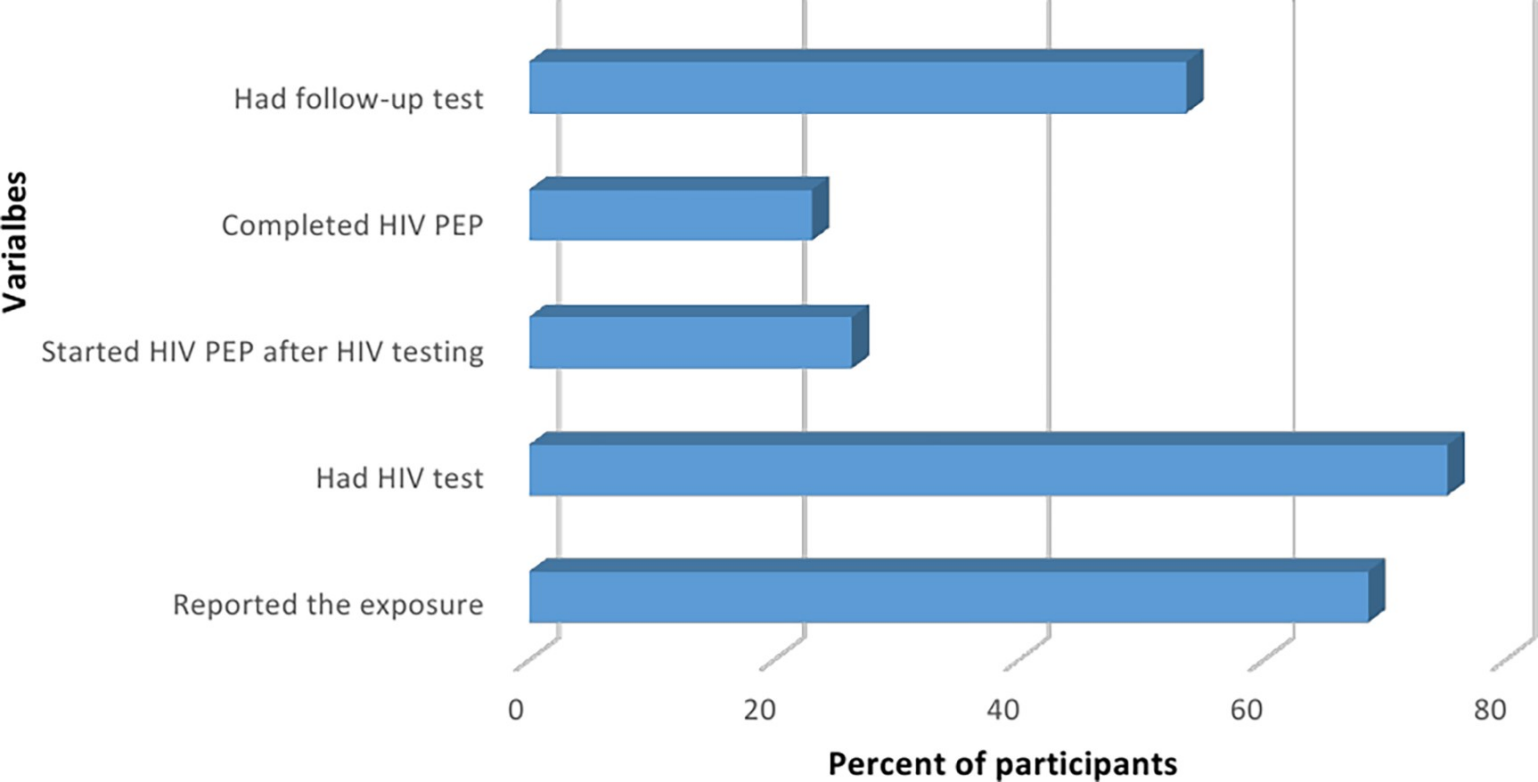
Utilization of HIV post exposure prophylaxis services among HCWs (n = 121).

Among 121 HCWs who had experienced occupational exposure, more than two thirds 83 (68.6%) reported the exposure incident to the management. Three quarters, 91 (75.2%) were tested for HIV and almost half, 65 (53.7%) had a follow-up HIV test. However, 32 (26.4%) started HIV PEP and 28 (23.1%) completed HIV PEP. Fig 1 summarizes these results:

## Discussion

Results from this study showed that more than half of HCWs are at risk of being infected with HIV through occupational exposure. Additionally, more than half had inadequate knowledge of HIV PEP and low utilization of HIV PEP following occupational exposures.

In this study the prevalence of occupational exposures was 50.6% among HCWs.The leading causes were blood splash followed by needle stick injuries. These findings suggest that most HCWs are at risk of acquiring HIV infection at their work sites. These findings are similar to those reported in Ethiopia[15]and Germany.[17] However, this prevalence of exposure is much higher compared to other findings from different settings, which showed the prevalence ranging from 19.2% to 30%[18][19]. The exposure differentials can be explained by study settings which may have an influence on the workload of participants, and the number of procedures performed by HCWs and low utilization of personal protective equipment[20–22] as reported in different areas of developing countries. However, the study was unable to capture time from exposure to reporting or level of significant exposure, which would be part of the risk assessment for PEP.

General knowledge on HIV PEP among HCWs was low; with more than half of the participants having inadequate knowledge. Most participants had inadequate knowledge of the drugs used for high risk. Other key areas were knowledge of definitions, criteria for offering certain HIV PEP regimens, knowledge of drugs used for low risk and knowledge of types of HIV PEP regimens according to level of risk. The observed high prevalence of occupational injuries and low knowledge of HIV PEP put HCWs more at risk of acquiring HIV and other infectious diseases due to occupational exposures. Similar findings on low knowledge on HIV-PEP have been reported in Nigeria-Agaba[23],Uganda[4]. and Nepal[24]. However, our study showed high proportion of participants were knowledgeable on how to use HIV PEP as well as the duration and the steps to be taken after an exposure. This finding was in accord to a study done in Nigeria showing about 93% of particiants had knowledge on HIV PEP timing and HIV PEP regimen for low risk exposure was found to be very high, 93% and 57% respectively.[4] Differences in these percentages can be explained by the lack of training on safety measures [24]and accessibility of information on safety measures post exposure.[4]

Almost three quarters of participants reported the availability of HIV PEP at their respective health facilities. However, testing for HIV, utilization and completion of HIV PEP following occupational exposures was very low compared to the knowledge of the availability of HIV PEP in the facilities. HCW’s low level of knowledge on HIV PEP could explain the low level of testing.

Contrary to these findings, in Nepal [24] and Ethiopia [15] no HCW was tested for HIV following occupational exposures. Low utilization of and completion of HIV PEP have been documented from different settings. For example, in Ethiopia, one third of exposed participants did not complete HIV PEP[13]. In South Africa, only one quarter completed HIV PEP[16],while in Nepal, none of HCWs used HIV PEP following occupational exposures.[24]

The most probable explanation for the observed low utilization and non-adherence to HIV PEP in this study can be due to the perceived side effects cause by HIV PEP medications. An alternative explanation could be the sero-negativity of the source person. The HCWs awareness regarding the negative results of the source person may influence the utilization and non-adherence to HIV PEP.

### Strength and limitations

The strength of this study is that it was conducted in a rural setting in Tanzania. Health care workers in rural settings of Tanzania serve more than 70% of the general population compared to their counterparts in urban settings. The study findings may assist health authorities in rural settings in improving the level of knowledge of HIV PEP, risks of exposure and utilization of HIV PEP in rural health facilities.

The study has some limitations. First, the nature of the study was cross sectional, which is limited in establishing the causal-pathways. Secondly, the study was conducted in rural settings; therefore the findings can not be generalized to the urban settings of Tanzania. Finally, most responses related to utilization of HIV PEP were self-reporting, hence there is a possibility of over reporting or underreporting. Despite the limitations of the study, the findings have contributed important information regarding the level of knowledge of HIV PEP, availability and utilization of HIV PEP among HCWs in Singida’s rural areas of Tanzania.

## Conclusions

Results from this study show that more than half of HCWs in Singida are at risk of being infected by HIV through occupational exposures. In addition, most have inadequate knowledge of HIV PEP and low utilization of HIV PEP following occupational exposures.

The findings highlight the need to improve HCW’s level of knowledge of HIV PEP, particularly in areas related to drugs used for high and low risk, definitions, and criteria for offering certain PEP regimens. Also emphases should be on the high risk of blood splash and needle stick injuries and the importance of testing post exposure, using and adherence to HIV PEP.

## Supporting information

S1 Questionnaire Used in the study. (DOCX)

S1 Database Lucina database_Excel. (XLSX)
