# Supplementary material for: Prevalence of occupational injuries and knowledge of availability and utilization of post exposure prophylaxis among health care workers in Singida District Council, Singida Region, Tanzania
Source: PLoS One. 2018 Oct 25;13(10):e0201695. doi: 10.1371/journal.pone.0201695 (PMC6201876; doi:10.1371/journal.pone.0201695)
Supplement: S1 Questionnaire — (DOCX) [file pone.0201695.s001.docx]

| Questionnaire for : **“ Prevalence of Occupational injuries and Utilization of HIV Post Exposure Prophylaxis among Health Care Workers at Singida District Council”**  Date of Interview:----/---/----  Health Facility: A: Hospital  B: Health Centre  C: Dispensary  Answer all questions  Circle the appropriate answer in column D.The question may have more than one answer. | | | | | | | | |
| --- | --- | --- | --- | --- | --- | --- | --- | --- |
| **Section A: SOCIAL DEMOGRAPHIC INFORMATION** | | | | | | | | |
| **A** | **B** | | **C** | | **D** | | **E** | |
| Q1: | Sex | | 1.Female  2.Male | | 01  02 | |  | |
| Q2: | What is your age (in years) | | Number: | | | |  | |
| Q3: | Marital Status | | 1.Married  2.Single  3.Divorced  4.Widow/Widower  5.Separated  6.Cohabit  7.Others (Specify)…….. | | 01  02  03  04  05  06  07 | |  | |
| Q4: | What is your cadre? | | 1. Medical Doctor  2. Surgeon  3. Assistant Medical Officer  4. Clinical Officer  5. Assistant Clinical Officer  6. Nurses(Registered/enrolled)  7. Lab personnel  8. Medical Attendants  9. Others (specify): | | 01  02  03  04  05  06  07  08  09 | |  | |
| Q5. | What is your education level? | | 1. Certificate  2. Diploma  3. Advance Diploma  4. Medical Degree  5. Master Degree | | 01  02  03  04  05 | |  | |
| Q6. | Which department are you working? | | 1. Pediatric ward  2. Medical ward  3. Surgical Ward  4. Obstetrics / Gynecology ward  5. Reproductive & Child Health Unit  6. Laboratory unit  7. Operating Theatre  8. Others (Specify): | | 01  02  03  04  05  06  07  08 | |  | |
| Q7. | What is your working experience? | | 1. Less than 5 years  2. 5-10 years  3. 10-15 years  4. Above 15 years | | 01  02  03  04 | |  | |
| Q8. | Who is the owner of this Health facility? | | 1. Government  2. Faith based organization  3. Non govt organization  4. Private for profit  5. Others (Specify): | | 01  02  03  04  05 | |  | |
| **Section B: PREVALENCE OF OCCUPATIONAL EXPOSURE** | | | | | | |  | |
| Q9. | | Have you ever been exposed to a needle prick or body splash or in contact with blood or body fluids? | | 1. Yes  2. No | 01  02 | | **If the answer is No, skip to Qn.13** | |
| Q10. | | Which type of accident/exposure did you experience? | | 1. Needle stick injury  2. Blood splash  3. Mucous splash | 01  02  03 | |  | |
|  |  |  |  | 4. Other body fluids (specify): | | | | |
| Q11. | | When was your last needle prick or body splash or in contact with blood or body fluids? | | 1. Within 3 months | | 01 | |  |
|  |  |  |  | 2. Within 6 months | | 02 | |  |
|  |  |  |  | 3. In the past one year | | 03 | |  |
| Q12. | | Did you report the accident? | | 1. Yes  2. No | | 01  02 | |  |
| **Section C: KNOWLEDGE ON OCCUPATIONAL INJURIES AND HIV-PEP SERVICES** | | | | | | | | |
| Q13. | | Define what is an occupational injuries | |  | | | | |
| Q14. | | Have you ever heard about HCWs who sustain needle stick injuries/exposures at workplace? | | 1. Yes  2. No  3. I don’t know | | 01  02  03 | |  |
| Q15. | | Define what is HIV-PEP medications? | |  | | | | |
| Q16. | | What are the sources of occupational injuries/exposures? | | 1. Needle stick injuries, blood, and body fluids | | 01 Yes  02 No | |  |
|  |  |  |  | 2. Vaginal secretions | | 01 Yes  02 No | |  |
|  |  |  |  | 3. Blood transfusions | | 01 Yes  02 No | |  |
|  |  |  |  | 4. Tears | | 01 Yes  02 No | |  |
|  |  |  |  | 5. Others (Specify): | | | | |
| Q17. | | HIV-PEP medications reduces the likelihood of HIV infection post exposure | | 1. Yes  2. No  3. I don’t know | | 01  02  03 | | |
| Qn 18. | | Recommended HIV-PEP regimen depends on the following: | | 1. Type of exposure | | 1. Yes  2. No  3. I don’t know | | |
|  |  |  |  | 2. HIV status of the source of exposure | | 1. Yes  2. No  3. I don’t know | | |
|  |  |  |  | 3. Viral load of the source of exposure | | 1. Yes  2. No  3. I don’t know | | |
| Qn 19. | | What are the regimens used for HIV-PEP medications according to risk levels? | | 1. Low risk-dual therapy (two drugs) | | 1. Yes  2. No  3. I don’t know | | |
|  |  |  |  | 2. Low risk-single therapy (one drugs) | | 1. Yes  2. No  3. I don’t know | | |
|  |  |  |  | 3. High-risk therapy (four drugs) | | 1. Yes  2. No  3. I don’t know | | |
|  |  |  |  | 4. High-risk triple therapy (three drugs) | | 1. Yes  2. No  3. I don’t know | | |
| Qn20. | | When is the appropriate time to start HIV-PEP medications post exposure? | | 1. Within 24 hours | | 1. Yes  2. No  3. I don’t know | | |
|  |  |  |  | 2. Within 72 hours | | 1. Yes  2. No  3. I don’t know | | |
|  |  |  |  | 3. Beyond 72 hours | | 1. Yes  2. No  3. I don’t know | | |
| Qn 21. | | What is the duration for HIV-PEP medications? | | 1. Within 72 hours | | 1. Yes  2. No  3. I don’t know | | |
|  |  |  |  | 2. Within 28 days | | 1. Yes  2. No  3. I don’t know | | |
|  |  |  |  | 3. Within 6 months | | 1. Yes  2. No  3. I don’t know | | |
| **Section D: AVAILABILITY OF HIV-PEP MEDICATIONS** | | | | | | | | |
| Qn.22 | | Are HIV-PEP medications available in this facility? | | 1. Yes  2. No  3. I don’t know | | 01  02  03 | | |
| Qn.23. | | Has this facility ever experienced unavailability of HIV-PEP medications? | | 1. Yes  2. No  3. I don’t know | | 01  02  03 | | |
| Qn 24. | | Are HIV-PEP medications services accessible all the time (i.e. day and night, weekends, and public holidays, etc.)? | | 1. Yes  2. No  3. I don’t know | | 01  02  03 | | |
| Qn 25. | | At this facility HIV-PEP medications are available at? | | 1. Care Treatment clinic (CTC) | | 1. Yes  2. No  3. I don’t know | | |
|  |  |  |  | 2. Pharmacy | | 1. Yes  2. No  3. I don’t know | | |
|  |  |  |  | 3. Matron’s office | | 1. Yes  2. No  3. I don’t know | | |
|  |  |  |  | 4. Referral to another facility | | 1. Yes  2. No  3. I don’t know | | |
|  |  |  |  | 5. Others (specify): | | | | |
| **Section E: UTILIZATION OF HIV-PEP MEDICATIONS** | | | | | | | | |
| Qn 26. | | Are HIV-PEP medications available in this facility? | | 1. Yes  2. No  3. I don’t know | | 01  02  03 | | |
| Qn 27. | | Is there someone to administer HIV-PEP medications 24 hours a day in this facility? | | 1. Yes  2. No  3. I don’t know | | 01  02  03 | | |
| Qn 28. | | Did you start HIV-PEP medications immediately post exposure? | | 1. Yes  2. No | | 01  02 | | |
| Qn 29. | | Did you report the injury/exposure immediately? | | 1. Yes  2. No | | 01  02 | | |
| Qn 30. | | Did you complete your HVI-PEP medications? | | 1. Yes  2. No | | 01  02 | | |
| **END OF THE QUESTIONNAIRE-THANK YOU FOR YOUR COOPERATION** | | | | | | | | |
